# Supplementary material for: Infant Food Security in New Zealand: A Multidimensional Index Developed from Cohort Data
Source: Int J Environ Res Public Health. 2019 Jan 21;16(2):283. doi: 10.3390/ijerph16020283 (PMC6352114; doi:10.3390/ijerph16020283)

**Figure S1.** Consort Diagram.

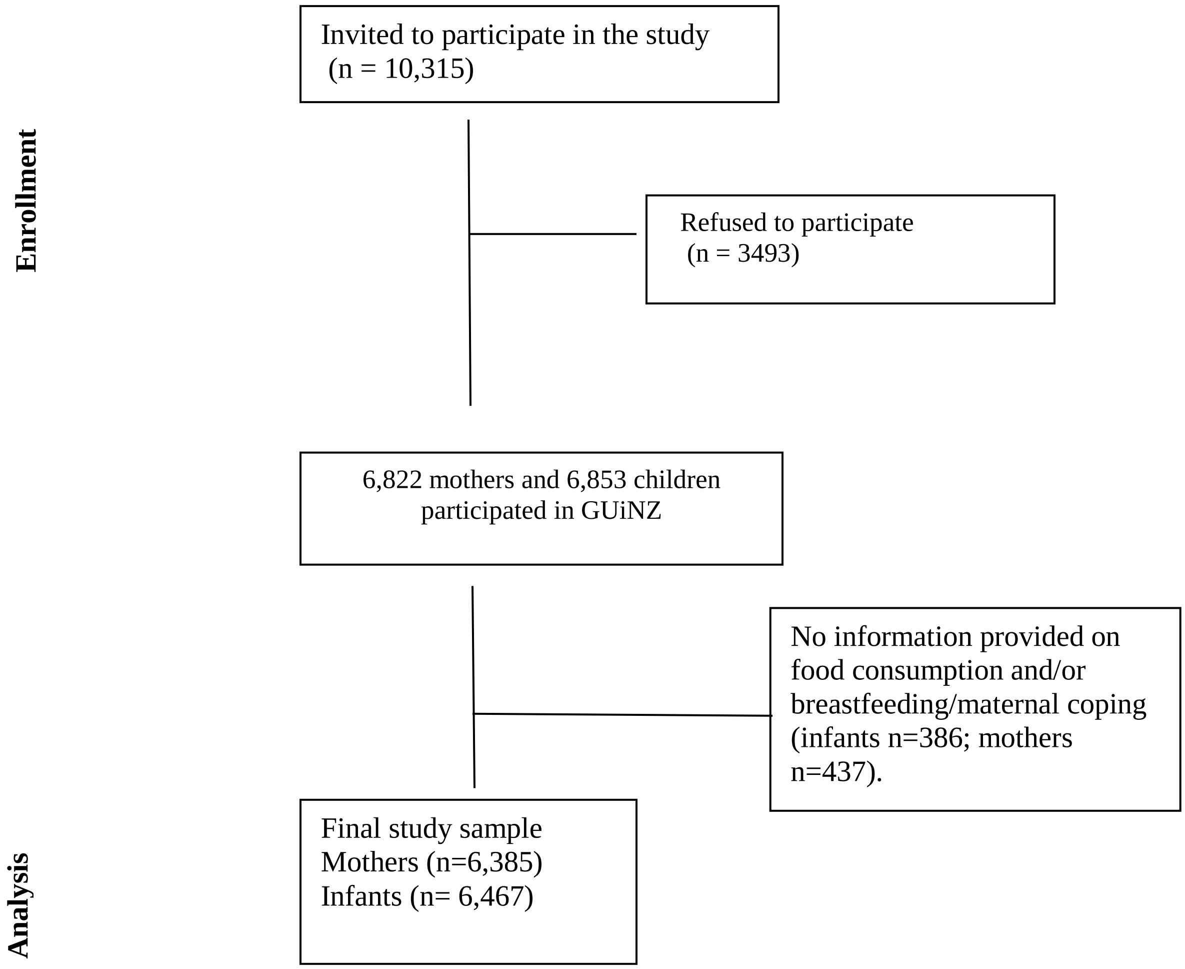

**Figure S2.** Histogram of Infant Food Security

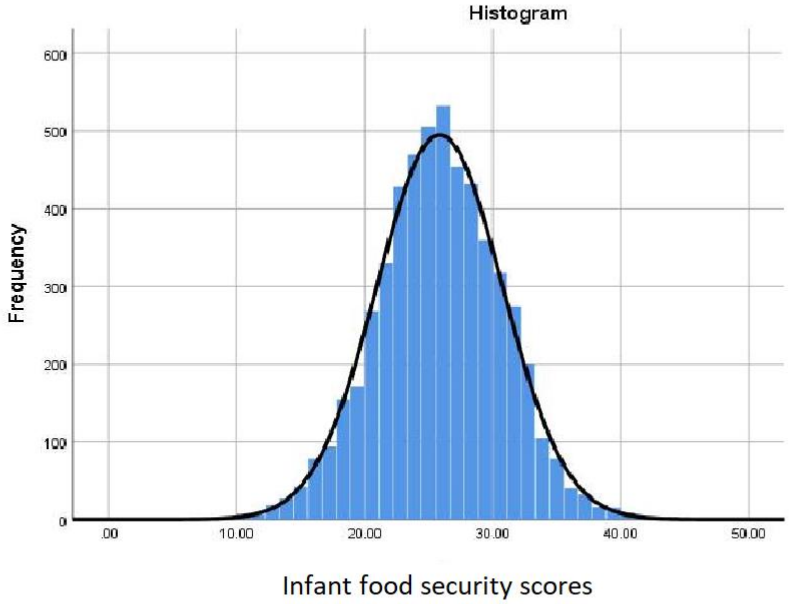

**Figure S3.** Normal Q-Q Plot of Infant Food Security Index

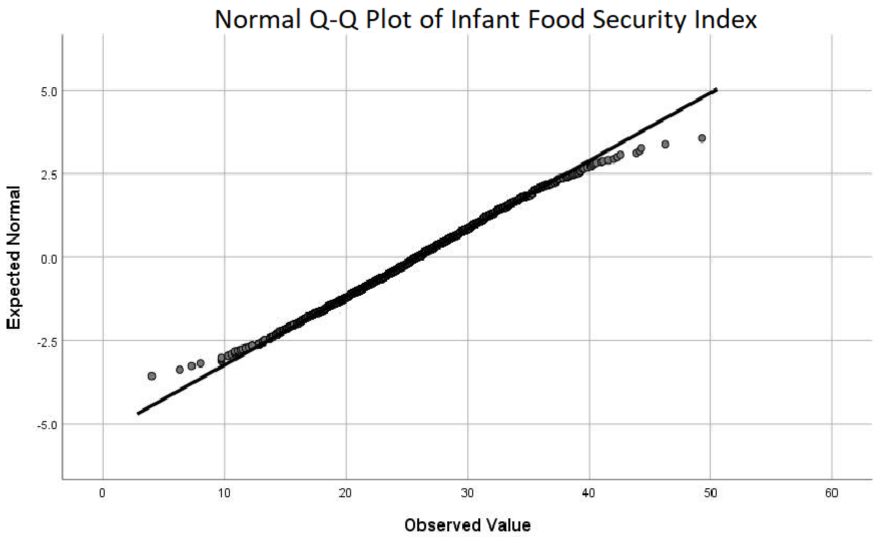

Supplement: Supplementary file 1 [file ijerph-16-00283-s001.pdf]
